# Role of Innate and Adaptive Cytokines in the Survival of COVID-19 Patients

**DOI:** 10.3390/ijms231810344

**Published:** 2022-09-07

**Authors:** Jorge Monserrat, Ana Gómez-Lahoz, Miguel A. Ortega, José Sanz, Benjamin Muñoz, Juan Arévalo-Serrano, José Miguel Rodríguez, Jose Maria Gasalla, Óscar Gasulla, Alberto Arranz, Jordi Fortuny-Profitós, Ferran A. Mazaira-Font, Miguel Teixidó Román, Carlos Martínez-A, Dimitri Balomenos, Angel Asunsolo, Melchor Álvarez-Mon, on behalf of the COVID-19 HUPA Group

**Affiliations:** 1 Department of Medicine and Medical Specialities, Faculty of Medicine and Health Sciences, University of Alcalá, 28801 Alcalá de Henares, Spain; 2 Ramón y Cajal Institute of Sanitary Research (IRYCIS), 28034 Madrid, Spain; 3 Cancer Registry and Pathology Department, Hospital Universitario Principe de Asturias, 28806 Alcalá de Henares, Spain; 4 Service of Internal Medicine and Immune System Diseases-Rheumatology, University Hospital Príncipe de Asturias (CIBEREHD), 28806 Alcalá de Henares, Spain; 5 Hospital Universitari de Bellvitge, Universitat de Barcelona, 08907 L’Hospitalet de Llobregat, Spain; 6 Department of Surgery, Medical and Social Sciences, Faculty of Medicine and Health Sciences, University of Alcalá, 28801 Alcalá de Henares, Spain; 7 Campus Nord, Universitat Politècnica de Catalunya, 08034 Barcelona, Spain; 8 Departament d’Econometria, Estadística I Economia Aplicada, Universitat de Barcelona, 08007 Barcelona, Spain; 9 Department of Immunology and Oncology, Centro Nacional de Biotecnología/CSIC, 28006 Madrid, Spain; 10 Department of Epidemiology and Biostatistics, Graduate School of Public Health and Health Policy, University of New York, New York, NY 10027, USA

**Keywords:** COVID-19, innate and adaptive cytokines, chemokines, growth factors, prognosis, biomarkers

## Abstract

SARS-CoV-2 is a new coronavirus characterized by a high infection and transmission capacity. A significant number of patients develop inadequate immune responses that produce massive releases of cytokines that compromise their survival. Soluble factors are clinically and pathologically relevant in COVID-19 survival but remain only partially characterized. The objective of this work was to simultaneously study 62 circulating soluble factors, including innate and adaptive cytokines and their soluble receptors, chemokines and growth and wound-healing/repair factors, in severe COVID-19 patients who survived compared to those with fatal outcomes. Serum samples were obtained from 286 COVID-19 patients and 40 healthy controls. The 62 circulating soluble factors were quantified using a Luminex Milliplex assay. Results. The patients who survived had decreased levels of the following 30 soluble factors of the 62 studied compared to those with fatal outcomes, therefore, these decreases were observed for cytokines and receptors predominantly produced by the innate immune system—IL-1α, IL-1α, IL-18, IL-15, IL-12p40, IL-6, IL-27, IL-1Ra, IL-1RI, IL-1RII, TNFα, TGFα, IL-10, sRAGE, sTNF-RI and sTNF-RII—for the chemokines IL-8, IP-10, MCP-1, MCP-3, MIG and fractalkine; for the growth factors M-CSF and the soluble receptor sIL2Ra; for the cytokines involved in the adaptive immune system IFNγ, IL-17 and sIL-4R; and for the wound-repair factor FGF2. On the other hand, the patients who survived had elevated levels of the soluble factors TNFβ, sCD40L, MDC, RANTES, G-CSF, GM-CSF, EGF, PDGFAA and PDGFABBB compared to those who died. Conclusions. Increases in the circulating levels of the sCD40L cytokine; MDC and RANTES chemokines; the G-CSF and GM-CSF growth factors, EGF, PDGFAA and PDGFABBB; and tissue-repair factors are strongly associated with survival. By contrast, large increases in IL-15, IL-6, IL-18, IL-27 and IL-10; the sIL-1RI, sIL1RII and sTNF-RII receptors; the MCP3, IL-8, MIG and IP-10 chemokines; the M-CSF and sIL-2Ra growth factors; and the wound-healing factor FGF2 favor fatal outcomes of the disease.

## 1. Introduction

Severe acute respiratory syndrome coronavirus 2 (SARS-CoV-2) is the agent responsible for the coronavirus disease 2019 (COVID-19), which emerged in Wuhan, China, in December 2019 and spread around the entire world [1]. In total, 519,105,112 cases and 6,266,324 fatalities had been documented across 239 nations and regions by 15 May 2022 [2].

SARS-CoV-2 is a single-strand RNA beta-coronavirus that expresses a spike (S) protein in its membrane that allows it to infect the epithelial cells of respiratory tissue through the angiotensin-converting enzyme (ACE-2) [3,4]. COVID-19 infection is characterized by complications of the lower respiratory system, including pneumonia, and the clinical presentations of the disease can be asymptomatic, moderate or severe [3]. Three main clinical stages of COVID-19 have been characterized: a viral phase response, a pulmonary phase and a systemic hyperinflammatory phase [5]. The first is the early phase of virus infection, which lasts about 3–6 days, with symptoms such as fever, cough and diarrhea. The second phase lasts about 5–13 days, in which there is lung involvement; this can be initiated without hypoxia, but patients end up developing hypoxia. The last phase starts at day 14 or sometimes earlier and is characterized by systemic hyperinflammation. The patients are usually reported to the hospital at the end of stage 1 or at the beginning of stage 2, when the immune system is already challenged [5]. The group at greater risk is composed, markedly, of those of advanced age or patients with previous pathologies or comorbidities. In older people, the increased risk is due to secondary immunodeficiency that develops as a consequence of impaired physiological function due to aging [3]. Other complications such as endocrine issues, digestive pathologies, hematological conditions, neoplastic conditions, virus infections, polytrauma, large burns, any pharmacological treatment that is strongly immunosuppressive, chemotherapy, radiotherapy, or exposure to environmental agents, along with any of the more than 450 congenital immunodeficiencies defined today, represent multiple risk factors that complicate the COVID-19 illness [3].

It has been described that patients with COVID-19 develop a status of the immune system in which cells are extremely activated and produce large amounts of cytokines, inducing a hyperinflammatory condition commonly referred to as a cytokine storm, mainly produced by the macrophage activation syndrome (MAS) [6,7,8]. These patients develop multiple organ failure and have a high mortality rate [6,7,8]. Therefore, several specific therapeutic strategies targeting cytokines such as IL-6 or IL-1 have been developed based on using drugs such as tocilizumab or anakinra [9]. The production of cytokines, chemokines and other soluble mediators of immune-inflammatory response appears to have a pivotal pathogenic role in the development of tissue lesions in different organs, especially in the lungs as main target of the severe infection [10]. They are also involved in the induction of cardiovascular complications and thrombotic events [11]. The cytokine storm has been related to the development of multiple organ failure and increased mortality rate of patients.

In general, soluble factors are a group of glycoproteins that play important roles in the development, cellular function and control of cells of the immune system and many other systems [12,13,14]. They are small secreted glycoproteins and proteolytically released from the cell surface, such as TNFα with autocrine, paracrine and endocrine roles usually produced upon the activation of cells of the immune system [15].

Many cytokines and soluble factors have been described, and several have been recognized as pivotal players in the immune system and human disease; in fact, many of them have been used as therapeutic tools [15]. Cytokines, unlike hormones, tend to be short-range protein mediators that carry out their activities at low concentrations, being able to perform pleiotropic, redundant and antagonistic functions.

Cytokines transmit their biological signals to responsive cells by interaction with specific high-affinity cell-surface receptors. These receptors are expressed at very low numbers, usually a few hundred to a few thousand per cell; however, they are very relevant in the immunoregulation of their target cells [12]. Some of these receptors are also soluble, playing roles in the regulation or neutralization of their cytokines, and notably, as in the case of IL-6R, they can amplify inflammatory responses. Soluble factors are classified according to their structural or receptor homology (five family groups) and gene proximity, and the classifications include cytokines, chemokines and growth factors [16,17]. However, the soluble factors can be classified according to different functional criteria. There is a group of cytokines and their receptors with established immune-modulating activity, with proinflammatory and anti-inflammatory effects, mainly produced by cells involved in innate or adaptive immune responses. The soluble factors that play a critical role in the traffic and tissue homing of leukocytes are the chemokines. Another group of soluble factors are growth factors that act on cells of the innate and adaptive immune systems. There are also cytokines directly involved in the regulation of the adaptive immune response. Finally, a group of soluble factors plays a relevant role in wound healing/tissue repair [18,19].

Chemokines are small, secreted proteins that mediate cell trafficking and lymphoid tissue development. They are classified into four groups according to the first two locations of cysteine in their protein sequences: the CC-, CXC-, C- and CX3C-chemokine families. Chemokines are extremely redundant, with many ligands binding to the same receptor, and vice versa [20]. Chemokines are essential in the amplification of inflammatory responses, the initial recruitment of cells of the innate immune system, and then that of cells of the adaptive immune system. The overexpression of chemokines mainly produced by monocytes and macrophages, such as IL-8, IP-10, MIP1 and MCP-1, is directly related to the severity of the disease in patients and is key for the infiltration of immune cells into the lung and, therefore, the severity of COVID-19 [6,20,21,22]. Wound-healing/repair growth factors are involved in angiogenesis and endothelial and tissue repair, as well as being associated with the differentiation of M2-type macrophages. The alteration of these soluble factors such as VEGF, PDGF-AA and PDGF-AB/BB also appears to be implicated in the progression of COVID-19 [23,24,25]. Therefore, in this study, cytokines are discussed on the basis of their biological role in COVID-19 patients rather than in terms of the families to which they belong.

There are a huge number of studies on the effects of cytokines on the pathology of COVID-19 and their role is being widely discussed [18,20,24,25,26,27,28,29,30,31,32,33]. In this study, we investigated the possible existence of a different pattern of variation in circulating levels of a wide number of soluble mediators of the immune-inflammatory response between patients with severe COVID-19 who survive or with a fatal outcome. We included in the study 286 severe COVID-19 patients and 40 sex- and age-matched healthy controls. We quantified the serum levels of 62 soluble factors classified into five groups according to their biological functions: (A) innate and common innate/adaptive proinflammatory and anti-inflammatory cytokines and receptors, (B) chemokines, (C) innate and adaptive growth factors, (D) adaptive cytokines and (E) wound-healing/repair growth factors.

## 2. Results

### 2.1. Characteristics of Patients

A total of 286 severe COVID-19 patients admitted to the hospital and 40 sex- and age-matched healthy controls were included in this study. Thus, 249 patients survived, of which 35 (14.05%) were admitted to the ICU; 37 patients died; 18 (48.65%) died inside the ICU and 19 (51.35%) died outside the ICU.

The demographic and clinical characteristics of the patients with severe COVID-19 included in the study are shown in Table 1. The patients with severe COVID-19 who progressed to exitus were significantly older than the survivors, but there was no significant difference according to sex. Additionally, significant differences in the Charlson and Elixhauser indices between both groups of patients were also detected (Charlson: *p* < 0.01; Elixhauser: *p* < 0.01). At hospital admission, patients with fatal outcome showed significantly reduced the blood oxygen saturation, significantly increased ferritin and D-Dimer serum levels and leukocytosis than those found in survivors.

We also studied the comorbidities of both groups of severe COVID-19 patients (hypertension, metabolic-endocrine diseases, heart disease, respiratory disease, kidney disease, autoimmune disease, hematologic and solid neoplasms, urinary tract infections, dementia and ulcerative colitis) (Table S1). The incidence of acute kidney failure and dementia was significantly higher in the group of non-surviving patients than that in that of the survivors.

### 2.2. Cytokine Results

For the analysis of the results, we classified the 62 soluble factors of the cytokines studied according to their major biological activities/roles/significance and chronology into five groups: (A) proinflammatory and anti-inflammatory cytokines mainly produced by cells involved in the innate and innate/adaptive immune responses and their receptors, (B) chemokines, (C) growth factors that act on cells of the innate and adaptive immune systems, (D) cytokines involved in the adaptive immune system response and (E) wound-healing/repair growth factors.

**Figure 1:**
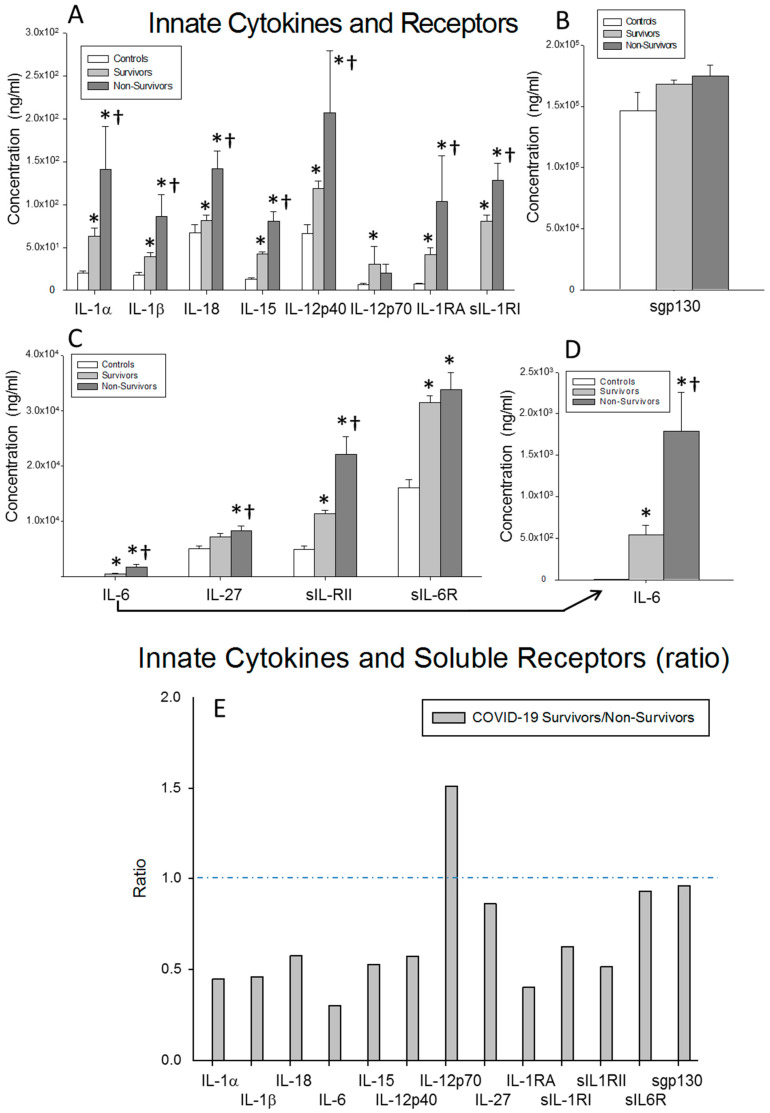
Results for inflammatory innate cytokines and soluble receptors. (**A**–**D**) Data represent the concentrations of the soluble factors indicated for (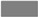) surviving COVID-19 patients, (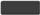) non-surviving COVID-19 patients and (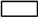) healthy controls. All the values are expressed as the means ± S.E.M.s *, *p* < 0.05 for surviving or non-surviving COVID-19 patients versus healthy controls. †, *p* < 0.05 for surviving versus non-surviving COVID-19 patients. (**E**) Data represent the ratios between the circulating levels in surviving and non-surviving COVID-19 patients of each soluble factor analyzed. The dotted line represents a reference line for the ratio equal to 1 between survivors and non-survivors.

First, we studied the circulating levels of IL-1α, IL-1β, IL-18, IL-15, IL-12p40, IL-12p70, IL-6, IL-27, IL-1Ra, IL-1RI, IL-1RII, IL-6R and sgp130, which are innate proinflammatory cytokines, and their receptors in survivors and non-survivors of severe COVID-19 and healthy controls. The surviving patients showed significantly lower circulating levels of the IL-1α, IL-1β, IL-18, IL-15, IL-12p40, IL-6 and IL-27 cytokines (*p* = 0.013, *p* = 0.005, *p* < 0.0001, *p* < 0.0001, *p* = 0.048, *p* < 0.0001 and *p* = 0.002, respectively) and the soluble receptors IL-1Ra, IL-1RI and IL-1RII (*p* = 0.008, *p* = 0.017 and *p* < 0.0001, respectively) than the non-survivors. Furthermore, the surviving and non-surviving severe COVID-19 patients showed significantly increased levels of circulating IL-1α, IL-1β, IL-6, IL-12p40, IL-15 and IL-18 (surviving patients vs. controls: *p* = 0.017, *p* = 0.048, *p* < 0.0001, *p* = 0.045, *p* < 0.0001 and *p* = 0.046, respectively; non-surviving vs. controls: *p* = 0.005, *p* = 0.002, *p*<0.0001, *p* = 0.049, *p* < 0.0001 and *p* = 0.012, respectively) compared to the healthy controls. The survivors showed significantly elevated levels of IL-12p70 (*p* = 0.043), while non-survivors showed significantly higher levels of IL-27 (*p* = 0.014), compared to the healthy controls. Interestingly, there were no significant differences in the sgp130 serum levels between the three groups of subjects (Figure 1A–D).

We also calculated the ratio of the serum concentration of each cytokine and cytokine receptor studied between the surviving and non-surviving severe COVID-19 patients. We found that the survivors showed increased ratios of IL-12p70 compared to the non-survivors. Interestingly, the ratios of the IL-6 receptor and spg130 were close to 1, but that of IL-6 was clearly decreased (Figure 1E).

**Figure 2:**
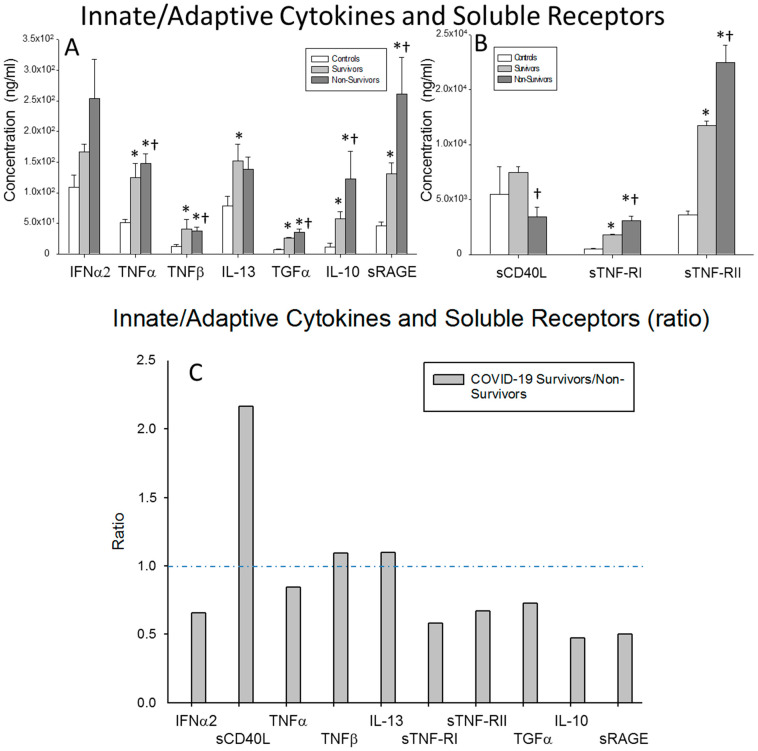
Inflammatory/anti-inflammatory innate and adaptive cytokines. (**A**,**B**) Data represent the concentrations of the cytokines indicated for (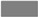) surviving COVID-19 patients, (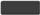) non-surviving COVID-19 patients and (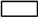) healthy controls. All the values are expressed as the means ± S.E.M.s *, *p* < 0.05 for surviving or non-surviving COVID-19 patients versus healthy controls. †, *p* < 0.05 for surviving versus non-surviving COVID-19 patients. (**C**) Data represent the ratios between the circulating levels in surviving and non-surviving COVID-19 patients of each soluble factor analyzed. The dotted line represents a reference line for the ratio equal to 1 between survivors and non-survivors.

Next, we studied the levels of pro-inflammatory and anti-inflammatory cytokines and their receptors involved in both innate/adaptive immune system responses. The surviving severe COVID-19 patients showed significantly lower circulating concentrations of TNFα, TGFα, IL-10, sRAGE, sTNF-RI and sTNF-RII compared to the non-survivors (*p* = 0.001, *p* = 0.031, *p* < 0.0001, *p* < 0.0001, *p* = 0.001 and *p* < 0.0001, respectively). However, the levels of sCD40L and TNFβ were significantly higher in survivors than in non-survivors (*p* = 0.002 and *p* = 0.019, respectively). The serum levels of TNFα, TNFβ, TGFα, IL-10, sRAGE, sTNF-RI and sTNF-RII in both groups of severe COVID-19 patients were significantly elevated compared to those found in the healthy controls (surviving patients vs. controls: *p* = 0.003, *p* = 0.008, *p* < 0.0001, *p* = 0.001, *p* < 0.0001, *p* < 0.0001 and *p* < 0.0001, respectively; non-surviving vs. controls: *p* < 0.0001, *p* = 0.002, *p* < 0.0001, *p* < 0.0001, *p* < 0.0001, *p* < 0.0001 and *p* < 0.0001, respectively) (Figure 2A,B).

The ratio of the serum concentration of sCD40L was clearly elevated and the ratios of TNFβ and IL-13 were slightly elevated in survivors compared to non-surviving severe COVID-19 patients. By contrast, the ratio of IL-10 was markedly reduced in the survivors (Figure 2C).

### 2.3. Serum Levels of Chemokines

**Figure 3:**
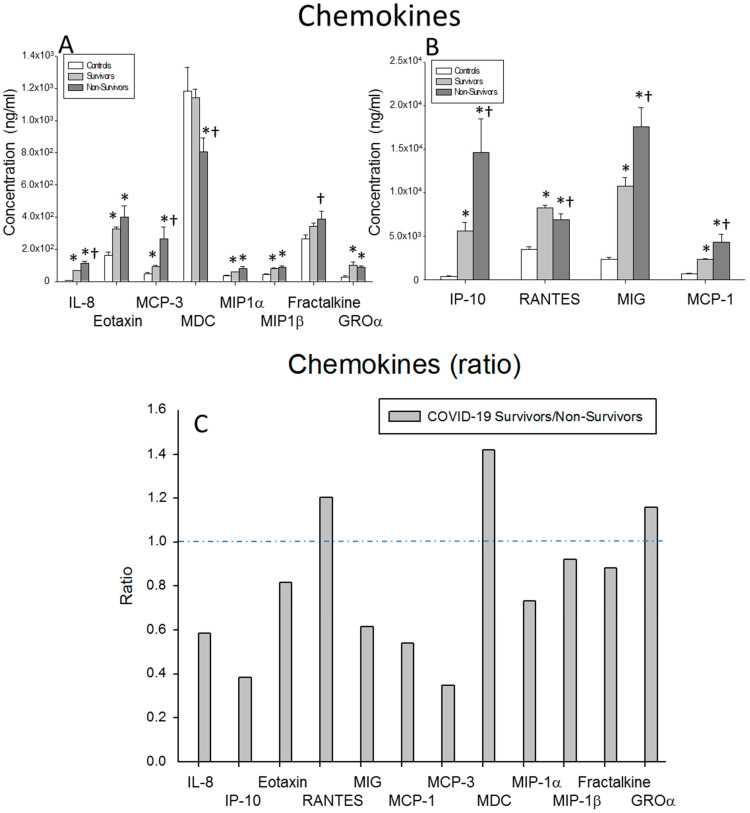
Chemokine results. (**A**,**B**) Data represent the concentrations of the chemokines indicated for (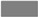) surviving COVID-19 patients, (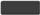) non-surviving COVID-19 patients and (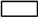) healthy controls. All the values are expressed as the means ± S.E.M.s *, *p* < 0.05 for surviving or non-surviving COVID-19 patients versus healthy controls. †, *p* < 0.05 for surviving versus non-surviving COVID-19 patients. (**C**) Data represent the ratios between the circulating levels in surviving and non-surviving COVID-19 patients of each chemokine analyzed. The dotted line represents a reference line for the ratio equal to 1 between survivors and non-survivors.

We studied the serum levels of the EOTAXIN, FRACTALKINE, GROα, IL-8, IP-10, MCP-1, MCP-3, MDC, MIG, MIP1α, MIP1β and RANTES chemokines in the surviving and non-surviving severe COVID-19 patients and healthy controls. Our results showed that the Il-8, MCP-3, FRACTALKINE, IP-10, MIG and MCP-1 levels were significantly decreased in the survivors compared to the non-survivors (*p* < 0.0001, *p* < 0.0001, *p* = 0.049, *p* < 0.0001, *p* < 0.0001 and *p* = 0.004, respectively). By contrast, the MDC and RANTES serum levels were significantly increased in the survivors compared to the non-survivors (*p* = 0.002 and *p* = 0.026, respectively). The serum concentrations of EOTAXIN, GROα, IL-8, IP-10, MCP-1, MCP-3, MIG, MIP1α, MIP1β and RANTES were significantly elevated in both groups of severe COVID-19 patients compared to the healthy controls (surviving patients vs. controls: *p* = 0.001, *p* < 0.0001, *p* < 0.0001, *p* = 0.010, *p* < 0.0001, *p* = 0.012, *p* < 0.0001, *p* = 0.003, *p* < 0.0001 and *p* < 0.0001, respectively; non-surviving vs. controls: *p* = 0.009, *p* < 0.0001, *p* < 0.0001, *p*<0.0001, *p* < 0.0001, *p* < 0.0001, *p* < 0.0001, *p* = 0.001, *p* < 0.0001 and *p* = 0.012, respectively) (Figure 3A,B).

The ratios of the serum concentrations of MDC, RANTES and GROα were markedly increased in the survivors compared to the non-surviving severe COVID-19 patients. By contrast, the ratio of MCP3 was markedly reduced in the survivors (Figure 3C).

### 2.4. Serum Levels of Growth Factors Acting on Hematopoietic and Immune System Cells (Innate and Adaptive Growth Factors)

**Figure 4:**
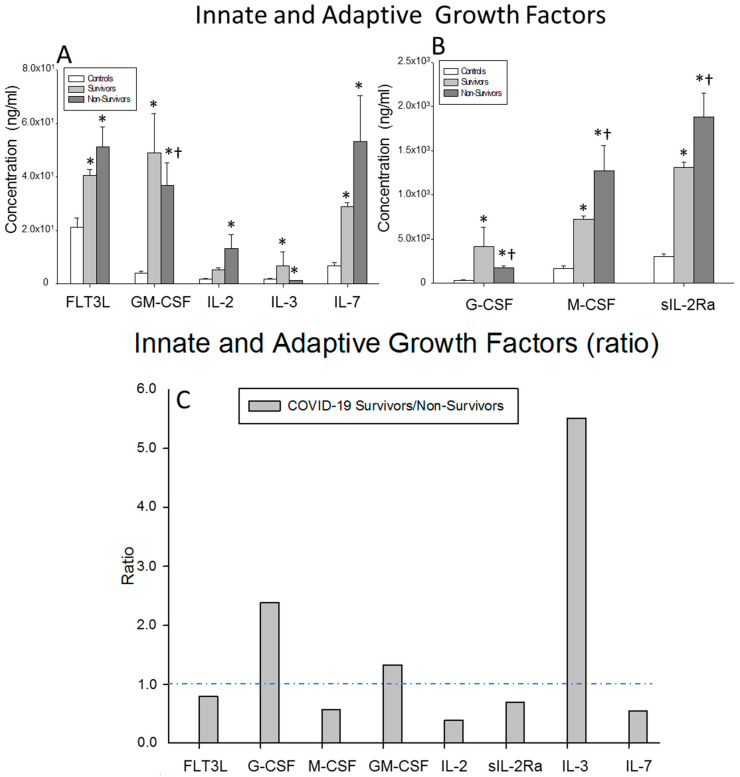
Results for innate and adaptive growth factors. (**A**,**B**) Data represent the concentrations of the growth factors indicated for (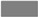) surviving COVID-19 patients, (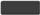) non-surviving COVID-19 patients and (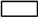) healthy controls. All the values are expressed as the means ± S.E.M.s *, *p* < 0.05 for surviving or non-surviving COVID-19 patients versus healthy controls. †, *p* < 0.05 for surviving versus non-surviving COVID-19 patients. (**C**) Data represent the ratios between the circulating levels in surviving and non-surviving COVID-19 patients of each growth factor analyzed. The dotted line represents a reference line for the ratio equal to 1 between survivors and non-survivors.

We investigated the circulating levels of the G-CSF, GM-CSF, M-CSF, IL-3, IL-7, FLT3L and IL-2 growth factors and soluble receptor sIL2Ra in the surviving and non-surviving severe COVID-19 patients and healthy controls. The M-CSF and sIL-2Ra levels were significantly decreased in the survivors compared to the non-survivors (*p* = 0.002 and *p* = 0.002, respectively). By contrast, the G-CSF and GM-CSF serum levels were significantly increased in the COVID-19 survivors compared to the non-survivors (*p* < 0.0001 and *p* = 0.044, respectively). The G-CSF, GM-CSF, M-CSF, IL-3, IL-7, FLT3L and sIL2Ra serum levels were significantly enhanced in both groups of patients with respect to the healthy controls (surviving patients vs. controls: *p* = 0.014, *p* = 0.040, *p* = 0.001, *p* = 0.046, *p* < 0.0001, *p* = 0.008 and *p* < 0.0001, respectively; non-surviving vs. controls: *p* < 0.0001, *p* = 0.030, *p* < 0.0001, *p* = 0.038, *p* < 0.0001, *p* = 0.006 and *p* < 0.0001, respectively) (Figure 4A,B).

The ratios of the serum concentrations of IL-3, GM-CSF and G-CSF were increased in the survivors compared to the non-surviving severe COVID-19 patients. By contrast, the ratios of IL-2, Il-7 and M-CSF were reduced in the survivors (Figure 4C).

### 2.5. Serum Levels of Cytokines Involved in the Adaptive Immune System Response

**Figure 5:**
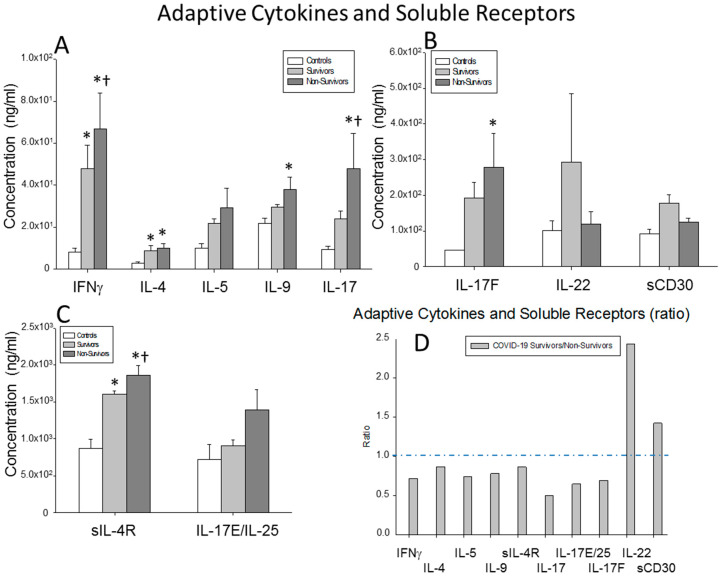
Adaptive cytokine results. (**A**–**C**) Data represent the concentrations of the cytokines and soluble receptors indicated for (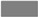) surviving COVID-19 patients, (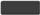) non-surviving COVID-19 patients and (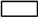) healthy controls. All the values are expressed as the means ± S.E.M.s *, *p* < 0.05 for surviving or non-surviving COVID-19 patients versus healthy controls. †, *p* < 0.05 for surviving versus non-surviving COVID-19 patients. (**D**) Data represent the ratios between circulating levels of surviving and non-surviving COVID-19 patients. The dotted line represents a reference line for the ratio equal to 1 between survivors and non-survivors.

We investigated the circulating levels of IFNγ, IL-4, sIL-4R, IL-5, IL-9, IL-17A, IL-17F, IL17-E/25, IL-22 and sCD30 in surviving and non-surviving severe COVID-19 patients and healthy controls. The IFNγ IL-17A and sIL-4R levels were significantly reduced in the survivors compared to the non-survivors (*p* = 0.020 *p* = 0.029 and *p* = 0.049, respectively). The IFNγ, IL-4 and sIL-4R serum levels were significantly enhanced in both groups of patients compared to the healthy controls (surviving patients vs. controls: *p* = 0.004, *p* = 0.001 and *p*<0.0001, respectively; non-surviving vs. controls: *p* = 0.001, *p* = 0.001 and *p* < 0.0001, respectively). In addition, the IL-9, IL-17A and IL-17F serum levels were significantly increased in the non-survivors compared to the healthy controls (*p* = 0.008, *p* = 0.030 and *p* = 0.019, respectively) (Figure 5A,B).

The ratios of the serum concentrations of IL-22 and sCD30 were enhanced in the survivors with respect to the non-surviving severe COVID-19 patients. Markedly, the ratio of IL-17A was reduced in the survivors (Figure 5C).

### 2.6. Serum Levels of Wound-Healing and Tissue-Repair Growth Factors

**Figure 6:**
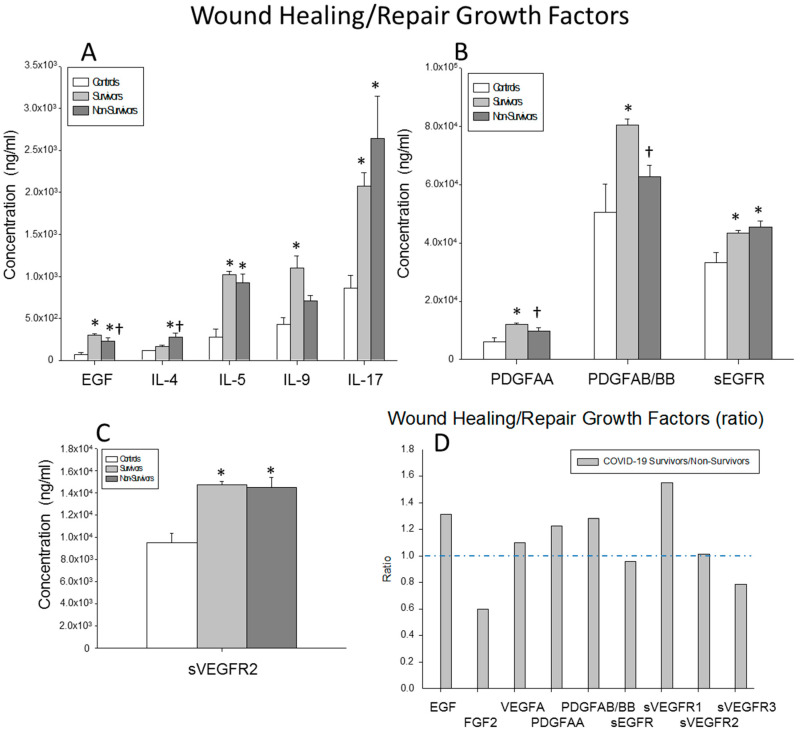
Results for wound-healing and repair growth factors. (**A**–**C**) Data represent the concentrations of the wound-healing and repair growth factors indicated for (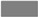) surviving COVID-19 patients, (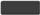) non-surviving COVID-19 patients and (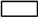) healthy controls. All the values are expressed as the means ± S.E.M.s *, *p* < 0.05 for surviving or non-surviving COVID-19 patients versus healthy controls. †, *p* < 0.05 for surviving versus non-surviving COVID-19 patients. (**D**) Data represent the ratios between the circulating levels in surviving and non-surviving COVID-19 patients of each wound-healing and repair growth factor analyzed. The dotted line represents a reference line for the ratio equal to 1 between survivors and non-survivors.

Finally, we studied the circulating levels of EGF, FGF2, VEGFA, sVEGFR1, sVEGFR2, SVEGFR3, PDGFAA, PDGFABBB and sEGFR. The surviving patients show significantly lower circulating levels of FGF2 than those found in the non-survivors (*p* = 0.001). By contrast, the serum levels of EGF, PDGFAA and PDGFABBB were significantly increased in the survivors compared to the non-survivors (*p* = 0.003, *p* = 0.008 and *p* = 0.001, respectively). Furthermore, the surviving and non-surviving severe COVID-19 patients showed significantly increased levels of circulating EGF, VEGFA, SVEGFR2, SVEGFR3 and sEGFR compared to the healthy controls (surviving patients vs. controls: *p* < 0.0001, *p* < 0.0001, *p* < 0.0001, *p* = 0.006 and 0.014, respectively; non-surviving vs. controls: *p* = 0.003, *p* < 0.0001, *p* = 0.002, *p* = 0.002 and *p* = 0.013, respectively). The survivors showed significantly elevated levels of sVEGFR1, PDGFAA and PDGFABBB compared to the healthy controls (*p* = 0.048, *p* = 0.002 and *p* = 0.006, respectively). The non-survivors showed FGF2 serum levels significantly elevated compared to those found in healthy controls (*p* = 0.003) (Figure 6A–C).

The ratios of the serum concentrations of EGF, VEGFA, PDGFGAA, PDGFAABB and sVEGFR1 were increased in the survivors compared to the non-survivors. By contrast, the ratio of FGF2 was decreased in the non-survivors (Figure 6C).

### 2.7. Survival Predictive Value Analysis of the Serum Soluble Factor Levels in Severe COVID-19 Patients

Finally, we analyzed the survival predictive value of all the cytokines, soluble receptors, chemokines, growth factors and wound-healing/repair factors studied in the severe COVID-19 patients by calculating their corresponding areas under the curve (ROCs), sensitivity and specificity and the statistical significance (Table S2).

**Figure 7:**
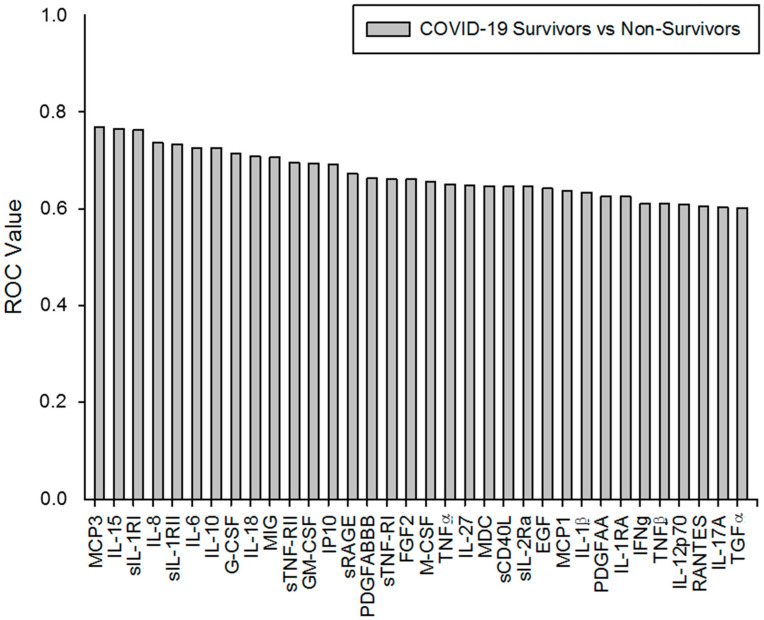
Soluble factors with significant predictive values. The data represented, indicated in the graphs with (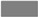), are ordered from the best to worst predictive values obtained (ROC values) from the first 34 soluble factors studied.

Of the 62 serum soluble factors studied, 34 presented significant predictive values for the survival of severe COVID-19 patients (Table 2). Taking into account the innate and innate/adaptive cytokines and soluble receptors analyzed in the serum, we found that severe COVID-19 with lower levels of IL-15, IL-6, IL-18, IL-27, IL-10, sIL-1RI, sIL-1RII and sTNF-RII, and higher levels of SCD40L showed significantly better predictive survival curves. We also observed that patients with lower serum concentrations of MCP3, IL-8, MIG and IP-10 and higher serum levels of the MDC and RANTES chemokines showed more significant predictive survival curves. The lower levels of the growth factors G-CSF and GM-CSF also showed relevant significant survival curves. On the other hand, with a smaller but significant predictive value, lower serum levels of the adaptive cytokines IFNγ and IL-17A also had predictive value in terms of survival in these patients. The lower serum levels of the repair factor FGF2 and the higher levels of PDGFABBB, EGF and PDGFAA had significant predictive value for a good prognosis in severe COVID-19 patients. Finally, the individual analysis of the soluble factors showed than the best area under the curve and significant survival predictive values were those of MCP-3, IL-15, sIL1-RI, IL-8, sIL-1RII, IL-6, IL-10, G-CSF, IL-18 and MIG (Figure 7).

## 3. Discussion

The interaction of SARS-CoV-2 with humans is a complex process with a marked involvement of the host immune system as well as different epithelial, mesenchymal and vascular cells of different organs and systems with special relevance for the respiratory system [4]. It has been shown that the immune-inflammatory response plays a critical role in the pathogenesis of COVID-19, with severe cases being associated with a marked dysregulation of cytokine production [5]. The relevance of the level of IL-6 as a biomarker of the prognosis of the disease has previously been demonstrated, and this has been identified as a target of immunotherapy [34]. Furthermore, the levels of other cytokines also appear to have relevant roles as prognostic markers of severe COVID-19 [18,25,26,27,35,36,37,38,39]. Cytokines are a complex network of molecules with different biologic activities promoting or suppressing immune-inflammatory responses and the lesioning or repair of tissues [12,13,14]. However, knowledge of the potential differential behavior of this wide cytokine network in severe COVID-19 and its clinical relevance for the progression of the disease in patients remain elusive.

We performed a comprehensive study of 62 soluble factors in patients with severe COVID-19 and centered our study on the critical first five days of admission at the hospital for patients who survived and non-survivors. IL-1, IL-6, IL-8 and TNFα are considered critical in the onset and maintenance of the immune-inflammatory response [8,16]. It has been proposed that SARS-CoV-2 stimulates innate immune system and induces IL-1 production and the subsequent downstream release of other proinflammatory cytokines, such as IL-6 and TNFα [4]. IL-1α and IL-1β are produced by immune cells such as monocytes, macrophages including alveolar macrophages and dendritic cells, and non-immune cells. IL-1α and IL1β act through the IL-1 receptor. The biological activity of IL-1 is modulated by its natural soluble inhibitors: sIL-1R1, sIL-1RII and IL1-RA [40,41].

The production of IL-1 through inflammasome activation is associated with that of IL-18 that favors IL-6, IL-8 and TNFα production [40,41]. IL-6 is considered pivotal for the onset of innate immune-inflammatory responses [16,17]. The biological activity of IL-6 is antagonized by a combination of sIL-6R and sgp130, which are soluble IL-6 receptors [42,43,44]. In agreement with previous studies, we found that severe COVID-19 patients had elevated serum levels of IL-1α, IL-1β IL-6 and IL-18 [18,25,26,32,37]. Our data show that the levels of these IL-1α, IL-1β IL-6 and IL-18 cytokines are significantly lower in severe COVID-19 survivors than in non-survivors. These results support the recognized prognostic value of the IL-6 serum levels in patients with severe COVID-19. Supporting this relevance of IL-6, although the non-surviving severe COVID-19 patients had significantly higher levels of IL-6, there were no significant differences between the two groups of patients in the levels of sIL-6R and sgp130, indicating limited control of IL-6 activity. Furthermore, circulating IL-6 and sIL-6R complexes (also called hyper-IL-6) may amplify inflammation, with the potential involvement of somatic non-immune cells that ubiquitously express the gp-130 receptor, through a non-classic form of activation called trans-signaling and in the absence of the regulatory mechanism of the sgp130 soluble factor [43,44]. Clearly, in the absence of sIL-6R, cells are not able to respond to IL-6; thus, treatment with anti-IL-6R has been shown to be efficient in the treatment of severe patients [45]. Interestingly, the levels of circulating IL-6 as well as those of sIL-6R were significantly reduced in the survivors compared to the non-survivors, but those of sgp130 were similarly increased in both patient subsets. These results support the recognized pathogenic relevance of IL-6 for the fatal outcome of the disease. Simultaneously, in severe COVID-19 non-survivors, the levels of sIL-1R1, sIL-1RII and IL-1RA are significantly higher than those in survivors. Thus, the differential pathogenic relevance of IL-1 serum levels for the two severe COVID-19 populations does not appear to be pivotal. The absence of a demonstrated impact of anti-IL-1 treatment in severe COVID-19 patients agrees with these findings [45,46,47]. Myelomonocytic cells are also the main producers of other cytokines with relevant immune-modulating effects such as IL-15, IL-12p40, IL-12p70 and IL-27 [48,49]. IL-15 is the most relevant cytokine regulating the proliferation and activation of NK cells and the cytotoxic-T-lymphocyte homeostasis [50]. IL-12p70 favors Th1 differentiation, which is antagonized by IL-12p40 [48]. IL-27 shows complex activity favoring Th1 and Treg differentiation [48]. Our results show that the levels of IL-12p70 are significantly increased in severe COVID-19 survivors compared to non-survivors, as are those of IL-12p40, IL-15 and IL-27. Thus, our results show reduced activation of myelomonocytic cells, with lower cytokine secretion, in patients with severe COVID-19 who survive. Increases in IL-12p40, IL-12p70, IL-15 and IL-27 in severe COVID-19 patients have previously been described [25,27,37]. The reduced levels of myelomonocytic cytokines found in severe COVID-19 survivors are not generalized because serum sCD40L, TNF-beta and IL-13 are increased. Interestingly, the differential outcome of severe COVID-19 is not associated with different levels of the pleotropic and antiviral IFNα2.

The pathogenesis of the immune-inflammatory response is associated with the migration of leukocytes to the target tissues [51,52]. Chemokines play an important role in the amplification of inflammation. Macrophages, dendritic cells, and other sentinel cells such as fibroblasts and endothelial cells release large amounts of chemokines that recruit neutrophils, monocytes, and dendritic cells to the damaged tissues [51,52]. IL-8 (CXCL8) and GROα (CXCL-1) attract neutrophils, MCP-1 (CCL-2) and MCP-3 (CCL-7) recruit mainly monocytes and macrophages, although they are also expressed by T and NK lymphocytes [19,51,52]. MCP-3 is able to attract dendritic cells and polymorphonuclear leukocytes. MIP1α, MIP1β and RANTES are responsible for attracting monocytes, polymorphonuclear cells and lymphocytes [19,51,52]. The presence of IFNγ induces the secretion of IP-10 and MIG, mainly by monocytes, plasmacytoid dendritic cells, endothelial cells and fibroblasts, recruiting to the site of infection IFNγ-producing lymphocytes such as Th1, Tc, NK and NKT cells, favoring their differentiation and proliferation, in addition to attracting more monocytes, macrophages and dendritic cells. Additionally, Tregs migrate in response to MDC (CCL22) secreted by macrophages and dendritic cells [19,51,52]. Eotaxin is released by vascular endothelial cells, stromal cells and immune cells, which recruit eosinophils to the lung [52]. Fractalkine is produced by alveolar macrophages and DCS and promotes the recruitment of monocytes and macrophages to the lung [53,54,55]. Our results show that the circulating levels of MCP-3, fractalkine, IP-10, MIG and MCP-1 were significantly lower in the severe COVID-19 surviving patients than in the non-survivors. Increased levels of IP-10 and MCP-1 have been associated with fatal outcomes [35,39]. Interestingly, the levels of MDC, GROα and RANTES were elevated in the survivors compared to the non-survivors. Thus, the levels of overproduction of chemokines appear to play a relevant role in the pathogenesis of severe COVID-19 and the outcome of the disease in these patients. Furthermore, the preferential secretion of MDC is observed in severe COVID-19 survivors. Interestingly, MDC favors tissue infiltration by Tregs and GROα favors the tissue infiltration of myeloid suppressor cells [20,21].

A relevant family of cytokines is those with growth-factor activity for hematopoietic and immune cells. M-CSF, G-CSF, GM-CSF, FLT-3L, IL-3 and IL-7 may act as hematopoietic factors but also have marked regulatory activity for cells involved in the innate and adaptive immune responses [17,56]. IL-2, IL-7 and IL-15 modulate the proliferation, differentiation and survival of the T cells, while IL-3 is responsible for the proliferation and survival of the lymphocyte progenitors [17]. IL-2 is necessary to induce the clonal expansion of T cells [17]. However, IL-2 may also upregulate FAS, inducing apoptosis and halting the T-cell immune response, and it may also favor the activation of Tregs for the secretion of anti-inflammatory cytokines such as IL-10 [57]. Additionally, circulating soluble IL-2 receptor (SIL-2R) regulates T-lymphocyte activation, decreasing the cellular response to IL-2. Thus, elevated levels of SIL-2R and IL-7 are associated with the severity of COVID-19 in patients and with their outcomes [19,37].

Our results show that the circulating levels of GM-CSF and G-CSF were significantly elevated but M-CSF was reduced in the surviving severe COVID-19 patients compared to the non-survivors. This reduced elevation of M-CSF agrees with the pattern of lower myelomonocytic cytokine production observed in severe COVID-19 survivors compared to non-survivors. Higher levels of GM-CSF have been associated with an increased risk of fatal outcomes in severe COVID-19 patients [19,38]. It is possible to suggest that their modulatory effects on peripheral blood and tissue granulocytes and myelomonocytic cells may be involved in the pathogenesis of the different clinical courses [58].

T lymphocytes play a critical role in the regulation of the adaptive response. The pattern of cytokine secretion by helper and cytotoxic T cells is regulated by different modulatory signals including cytokines involved in the innate response [59]. IFNγ plays a critical role in the activation of cytotoxic T cells and NK cells [15]. IL-4, IL-5 and IL-9 show pivotal regulatory effects in B-lymphocyte differentiation and eosinophil activity [18]. The IL-17 cytokine family has a modulatory effect on neutrophils. IL-22 is a cytokine with effects on non-immune cells and is involved in tissue-repair mechanisms [18]. Our results show that surviving severe COVID-19 patients have lower circulating levels of IFNγ, IL-17A and sIL-4R than those with fatal outcomes. However, there were no differences in the serum levels of IL-4, IL-5, IL-9, IL-17F and IL-22 between the two groups of patients. Controversial results have been described regarding the levels of these cytokines in patients with severe COVID-19 [19,24,25,26,27,28,29,30,31,32,33]. These data suggest that the elevated levels of IFNγ and IL-17A might induce an overactivation of cytotoxic lymphocytes and neutrophils, which could play a relevant pathogenic role in the fatal progression of the disease [3,60,61,62].

Soluble factors are also involved in homeostasis and the repair of tissue damage. Elevations in the EGF, FGF2, VEGFA, sVEGFR1, sVEGFR2, sVEGFR3, PDGFAA, PDGFABBB and sEGFR repair factors have been observed in patients with COVID-19. Our data demonstrate that significantly increased levels of EGF, PDGFAA and PDGFABBB are associated with better courses of the disease, indicating the relevance of angiogenesis for the favorable outcome of the disease.

The different pattern of alteration of the levels of soluble factors found in survivors and non-survivors with COVID-19 led us to analyze the prognostic significance of each of the 62 circulating soluble factors studied. Our data show that low levels of the IL-15, IL-6, IL-18, IL-27, IL-10, sIL-1RI, sIL1RII, sTNF-RII, MCP3, IL-8, MIG, IP-10, G-CSF and GM-CSF soluble factors and high levels of sCD40L, MDC and RANTES have prognostic value for good course of the disease and survival.

Taken together, in our study carried out on a large population of patients with severe COVID-19 with simultaneous analysis of the serum levels of soluble factors, our data indicate that the response pattern of the immune-inflammatory system is associated with different clinical courses in COVID-19 patients (Table S3). Lower activation of the innate response, with reduced elevation of IL-1a, IL-1b, IL-15, IL-6, IL-18, IL-27, IL-10, IL-12p40, TNFα, TNF-RI, TNF-RII, IL-1RA, IL-1-RI and IL-1-RII and increased sCD40L, promotes a favorable prognosis for the patient. Additionally, a reduction in the adaptive response of T lymphocytes, with lower circulating levels of IFNγ, IL-17A and sIL-4R, shows good prognostic value. The traffic and homing of leukocytes with differential patterns of circulating chemokine levels, with lower increases in IL-8, IP-10, MCP-1, MCP-3, MIG and fractalkine and decreases in MDC and RANTES, are also associated with good clinical outcomes.

In this work, the quantification of circulating soluble mediators was performed in serum samples. This study strategy may have a limitation due to the potential impact of the possible release of some chemokines such as RANTES, IL-8, GROα, MIP1α or MCP-3, growth factors such as VEGF, FGF, PDGF and pro-inflammatory cytokines such as TNFα or TNFβ by platelets during the clot formation process [63]. However, the study of the serum concentrations might also reflect the potential involvement of platelets in the pathogenesis of severe COVID-19 and its fatal outcome. Future studies should perform a logistic regression between cytokines as well as associations with clinical data. However, this is one of the pathophysiological studies that has the largest sample size and that allows us to understand the mechanisms in this important and dynamic disease.

## 4. Materials and Methods

### 4.1. Study Cohorts and Inclusion and Exclusion Criteria

This study was designed as an observational, analytical, retrospective cohort study with a longitudinal follow-up. The study population consisted of 286 consecutive patients diagnosed with COVID-19 (infected with the original SARS-CoV-2 Wuhan-1 strain) according to the criteria established by the World Health Organization based on the results of a real-time reverse-transcription polymerase chain reaction (RT-qPCR) test on a nasopharyngeal sample [31]. Blood samples were submitted to the Biochemistry Service of the Hospital Universitario Principe de Asturias during the first five days of the Hospital admission. The inclusion criteria for patients admitted to the Hospital Universitario Principe de Asturias (HUPA) were (1) a respiratory rate ≥ 30 breaths/min, (2) oxygen saturation SpO_2_ ≤ 94% while breathing ambient air, and (3) opacities detected in a chest X-ray as defined by the Diagnosis and Treatment Protocol for Novel Coronavirus Pneumonia (6th interim edition) [64]. We obtained demographic data, as well as information about comorbidities, from electronic health records.

Standard treatment (ST) and clinical management were carried out according to established protocols. On admission, patients received oxygen support through a low-flow nasal cannula to maintain SpO_2_ > 90%. Patients with increased oxygen needs were switched to a high-flow oxygen mask (Venturi mask up to 50% FiO_2_). Mechanical ventilation (MV) was provided only to patients admitted to the ICU. Thus, 286 blood samples were collected within the first five days of hospital admission. Sera from 40 age-matched healthy individuals were used as controls.

### 4.2. Study Protocol and Assays of Cytokine Serum Levels

Blood samples were obtained in sterile clotting tubes from peripheral blood using ante-cubital venipuncture. To obtain the serum, these samples were centrifuged at 2000× *g* for 20 min at 4 °C, aliquoted, identified and labeled, and frozen at −80 °C until quantification. Then, they were thawed and evaluated using two high-sensitivity human panel MILLIPLEX^®^ kits (MerkMillipore, Boston, MA, USA) to simultaneously measure the following: panel A: soluble (s) sCD40L ligand (L), EGF, EOTAXIN, FGF2, FLT3L, FRACTALKINE, G-CSF, GM-CSF, GROα, IFNα2, IFNα, IL-1α, IL-1α, IL-1RA, IL-2, IL-3, IL-4, IL-5, IL-6, IL-7, IL-8, IL-9, IL-10, IL-12p40, IL-12p70, IL-13, IL-15, IL-17A, IL-17E/IL-25, IL-17F, IL-18, IL-22, IL-27, IP-10, MCP-1, MCP-3, M-CSF, MDC, MIG, MIP1α, MIP1α, PDGFAA, PDGFABBB, RANTES, TGFα, TNFα, TNFα and VEGFA; and panel B: sCD30, sEGFR, sgp130, sIL-1RI, sIL-1RII, sIL-2Ra, sIL-4R, sIL-6R, sRAGE, sTNF-RI, sTNF-RII, sVEGFR1, sVEGFR2 and sVEGFR3. The kits were used for the assays following the manufacturer’s instructions, obtaining the results using the Luminex MAGPIX^®^ System (MerkMillipore, Boston, MA, USA). The results were analyzed using the MILLIPLEX^®^ Analyst 5.1 software (MerkMillipore, Boston, MA, USA). The sensitivity limits for the 62 tested circulating soluble factors are described in Table S2.

Briefly, for the studies of cytokines using the Luminex system, a pool of microspheres was used, each one encoded with a percentage of red and infrared fluorescence emission depending on the analyte to be studied. These were incubated for 16–18 h with the antigen to bind to the capture antibody for each microsphere in a 96-well plate. After incubation, the biotinylated detection antibody for each cytokine was added. Finally, a streptavidin–phycoerythrin complex (Strep-PE) was added, which bound to the detection antibody, and the plate was read in the MAGpix^®^ System (MerkMillipore, Boston, MA, USA). The results were obtained using 62 standard curves, and the concentration of each cytokine of interest was obtained based on the mean fluorescence intensity (MFI) and the standard curves.

### 4.3. Statistical Analysis

Analyses were performed using the SPSS 22 software (SPSS-IBM, Armonk, NY, USA). Since most of the variables did not fulfill the normality hypothesis, the Mann–Whitney U-test for non-parametric data was used to analyze differences between groups. The significance level was set at *p* < 0.05. To assess the value of baseline circulating soluble factors as predictors of survival, a receiver operating characteristic (ROC) curve analysis was performed, and the respective areas under the curve (AUCs) were determined. The best predictive cut-off value was defined as that which resulted in the highest product of the sensitivity, specificity, positive predictive value (PPV) and negative predictive value (NPV). The significance level was set at *p* < 0.05.

### 4.4. Ethics and Approval

This study was conducted according to basic principles of ethics (autonomy, harmlessness, benefit and distributive justice). The protocol was in line with the standards of Good Clinical Practice and the principles of the last Declaration of Helsinki (2013) and the Oviedo Convention (1997). Ethics committee approval was obtained from the University Hospital Príncipe de Asturias (HUPA-04062020).

## 5. Conclusions

The circulating levels of growth factors that act on hematopoietic and immune-system cells and wound-healing and tissue-repair growth factors play a relevant role in the course of the disease. High levels of GM-CSF, G-CSF, EGF, PDGFAA and PDGFABBB and low levels of M-CSF, FGF2 and sIL-2Ra are associated with a good prognosis. These data suggest that, in the treatment of COVID-19, there may be more therapeutic targets, and it is possible that a strategy of greater immunomodulation can prevent the fatal progression of the disease.

Our results clearly show a differential pattern of cytokine serum levels between patients with severe COVID-19 who survive and those with fatal outcomes.

## Figures and Tables

**Table 1:** Description of the COVID-19 patients included in the study. Demographic and clinical characteristics (means and standard deviations) of the patients who survived versus those who did not.

	Survivors (249)	Non-Survivors (37)	Total	*p*-Value
Age ( years), mean (SD)	62.95 (12.35)	70.32 (12.19)	63.90 (12.55)	<0.01
Gender (male)	63.86%	72.97%	65.03%	0.27
**Oxygen saturation on Hospital admission**				<0.01
Extremely low (<80%)	6.02%	18.92%	7.69%	
Low (80–89%)	12.45%	32.43%	15.03%	
Medium (90–94%)	57.83%	48.65%	56.64%	
Normal (>94%)	23.69%	0%	20.63	
No. Leucocytes, mean (SD), (n°/μL)	8258.94 (4822.31)	13,272.58 (11980.6)	8910.26 (6429.82)	<0.01
Ratio Lymphocytes/Leucocytes, mean (SD)	0.18(0.11)	0.12(0.15)	0.17(0.12)	<0.01
D-Dimer, mean (SD), (ng/mL)	3296.63 (13,408.49)	9500.24(11,480.64)	4086.18 (13,324.76)	<0.01
Ferritin, mean (SD), (ng/mL)	787.89 (644.57)	1405.95(1136.21)	865.63 (751.05)	<0.01
Charlson Index	0.93 (1.40)	1.72 (1.40)	1.03 (1.43)	<0.01
Elixhauser Index	1.89 (1.69)	2.91 (1.86)	2.02 (1.74)	<0.01
**Commorbidities**				
Acute Kidney failure	8.43%	27.03%	10.84%	<0.01
Dementia	0.80%	8.11%	1.75%	<0.01

**Table 2:** Survival predictive analysis of the best serum soluble factor levels included in this study in severe COVID-19 patients.

**1. Proinflammatory and Anti-Inflammatory Cytokines of the Innate and Innate/Adaptive Immune Systems**					
**Innate**	**Criterion**	**Sensitivity**	**Specificity**	**Area under the ROC Curve**	**Significance Value (*p*)**
IL-15	≤44.7	71.67	76.74	0.765	0.0001
sIL-1RI	≤129.2	90.24	62.5	0.764	0.0112
sIL-1RII	≤12544	75.77	65.12	0.733	0.0001
IL-6	≤158.73	74.74	67.44	0.726	0.0001
IL-18	≤68.02	61.38	79.07	0.708	0.0001
IL-27	≤5701	58.16	69.77	0.649	0.0019
IL-1β	≤42.24	81.44	41.86	0.634	0.0055
IL-1RA	≤14.7	55.94	67.44	0.625	0.0095
IL-12p70	≤4.94	42.17	81.82	0.609	0.048
**Innate/adaptive**					
IL-10	≤19.54	53.28	86.05	0.726	0.0001
sTNF-RII	≤11487	58.7	74.42	0.696	0.0001
sRAGE	≤99.58	66.8	63.41	0.673	0.0004
sTNF-RI	≤2476	82.07	48.84	0.661	0.0008
TNFα	≤102.75	67.69	58.14	0.651	0.0016
sCD40L	>3781	53.58	76.74	0.646	0.0004
TNFβ	≤21.52	66.78	55.81	0.611	0.0214
TGFα	≤20.29	53.42	69.77	0.602	0.0347
**2. Chemokines**					
MCP3	≤71.67	68.26	76.74	0.769	0.0001
IL-8	≤77.12	70.99	74.42	0.737	0.0001
MIG	≤6689	47.96	86.05	0.707	0.0001
IP-10	≤433.19	48.81	83.72	0.691	0.0001
MDC	>628	76.19	48.84	0.647	0.0004
MCP1	≤2752	78.23	53.49	0.637	0.0044
RANTES	>3371	94.88	32.56	0.605	0.0153
**3. Innate and Adaptive Growth Factors**					
G-CSF	≤58.68	54.47	89.74	0.715	0.0001
GM-CSF	≤9.81	45.1	100	0.694	0.0502
M-CSF	≤927.52	72.4	53.66	0.655	0.0015
sIL-2Ra	≤979.22	44.86	79.07	0.646	0.0023
**4. Adaptive Cytokines**					
IFNγ	≤25.15	70.98	52.38	0.611	0.023
IL-17A	≤10.42	48.24	74.42	0.603	0.0327
**5. Wound-Healing/Repair Growth Factors**					
PDGFABBB	>63788	64.29	65.12	0.664	0.0001
FGF2	≤112.23	45.55	83.72	0.661	0.0007
EGF	>169.49	65.64	65.12	0.642	0.0006
PDGFAA	>8732	65.99	58.14	0.626	0.0029

## Data Availability

The data used to support the findings of the present study are available from the corresponding author upon request.

## Supplementary Materials

The following supporting information can be downloaded at: https://www.mdpi.com/article/10.3390/ijms231810344/s1.
