# Supplementary material for: Role of Innate and Adaptive Cytokines in the Survival of COVID-19 Patients
Source: Int J Mol Sci. 2022 Sep 7;23(18):10344. doi: 10.3390/ijms231810344 (PMC9499609; doi:10.3390/ijms231810344)
Supplement: Supplementary file 1 [file ijms-23-10344-s001.zip › ijms-1846435-supplementary.pdf]

Table S1. Description of the COVID-19 patients included in the study. Demographic and clinical characteristics (means and standard deviations) of the patients who survived versus those who did not.

	**Survivors (249)**	**Non-Survivors(37)**	**Total**	***p*-value**
Age (years), mean (SD)	62.95 (12.35)	70.32 (12.19)	63.90 (12.55)	<0.01
Gender (male)	63.86%	72.97%	65.03%	0.27
**Oxygen saturation on Hospital admission**				<0.01
Extremely low (<80%)	6.02%	18.92%	7.69%	
Low (80-89%)	12.45%	32.43%	15.03%	
Medium (90-94%)	57.83%	48.65%	56.64%	
Normal (>94%)	23.69%	0%	20.63	
No. Leucocytes, mean (SD),(nº/µl)	8258.94(4822.31)	13272.58(11980.6)	8910.26(6429.82)	<0.01
Ratio Lymphocytes/Leucocytes, mean (SD)	0.18(0.11)	0.12(0.15)	0.17(0.12)	<0.01
D-Dimer, mean (SD), (ng/ml)	3296.63(13408,49)	9500.24(11480.64)	4086.18(13324.76)	<0.01
Ferritin, mean (SD), (ng/ml)	787.89(644.57)	1405.95(1136.21)	865.63(751.05)	<0.01
Charlson Index	0.93 (1.40)	1.72 (1.40)	1.03 (1.43)	<0.01
Elixhauser Index	1.89 (1.69)	2.91 (1.86)	2.02 (1.74)	<0.01
**Commorbidities**				
**Hypertension**				
Uncomplicated	36.55%	37.84%	36.71%	0.88
Complicated	6.02%	13.51%	6.99%	0.09
**Metabolic-endocrine diseases**				
Diabetes	22.09%	32.43%	23.43%	0.16
Uncomplicated	14.68%	24.32%	16.08%	0.14
Complicated	7.23%	8.11%	7.34%	0.84
Hypothyroidism	7.23%	5.41%	6.99%	0.68
Obesity	9.24%	16.22%	10.14%	0.19
**Heart diseases**				
Pulmonary Circulation Disorders	9.64%	16.22%	10.49%	0.22
Ischemic heart disease	5.62%	8.11%	5.94%	0.55
Congestive Heart Failure	5.62%	13.51%	6.64%	0.07
**Respiratory diseases**				
Asthma	8.43%	2.70%	7.64%	0.22
COPD (Chronic obstructive pulmonary disease)	5.62%	13.51%	6.64%	0.07
**Kidney diseases**				
Acute Kidney failure	8.43%	27.03%	10.84%	<0.01
Chronic Kidney disease	5.22%	10.81%	5.94%	0.18
**Autoimmune diseases**				
Reumathoid arthritis	5.22%	0,0%	4.55%	
Spondiloarthritis	1.61%	2.70%	1.75%	0.63
Psoriasis	1.20%	0,0%	1.05%	
Vasculitis	5.22%	8.11%	5.59%	0.47

**Hematologic neoplasms**				
Leukemia	0.40%	2.70%	0.7%	0.11
Lymphoma	0.80%	0,0%	0.70%	
Multiple myeloma	0.80%	0,0%	0.70%	
**Solid neoplasms**				
Bowel Cancer	0.40%	2.70%	0.70%	0.12
Breast Cancer	0,0%	0,0%	0,0%	
Lung Cancer	0.40%	0,0%	0.35%	
**Others**				
**Urinaty Tract Infection (UIT)**	3.61%	2.70%	3.5%	0.77
**Dementia**	0.80%	8.11%	1.75%	<0.01
**Ulcerative Colitis**	0.4%	0,0%	0.35%	

Table S2. Survival predictive analysis of the best serum soluble factor levels included in this study in severe COVID-19 patients.

1. Proinflammatory and anti-inflammatory cytokines of the innate and innate/adaptive immune systems

Innate	Criterion	Sensitivity	Specificity	Area under the ROC Curve	Significance Value (*p*)
IL-15	≤44.7	71.67	76.74	0.765	0.0001
sIL-1RI	≤129.2	90.24	62.5	0.764	0.0112
sIL-1RII	≤12544	75.77	65.12	0.733	0.0001
IL-6	≤158.73	74.74	67.44	0.726	0.0001
IL-18	≤68.02	61.38	79.07	0.708	0.0001
IL-27	≤5701	58.16	69.77	0.649	0.0019
IL-1β	≤42.24	81.44	41.86	0.634	0.0055
IL-1RA	≤14.7	55.94	67.44	0.625	0.0095
IL-12p70	≤4.94	42.17	81.82	0.609	0.048
IL-1α	≤59.93	80.37	39.02	0.579	0.1109
sIL-6R	≤30708	61.3	51.16	0.536	0.4465
sgp130	≤226276	87.37	27.91	0.53	0.5313
IL-12p40	>47.4	70.79	44.19	0.515	0.757
**Innate/ adaptive**					
IL-10	≤19.54	53.28	86.05	0.726	0.0001
sTNF-RII	≤11487	58.7	74.42	0.696	0.0001
sRAGE	≤99.58	66.8	63.41	0.673	0.0004
sTNF-RI	≤2476	82.07	48.84	0.661	0.0008
TNFα	≤102.75	67.69	58.14	0.651	0.0016
sCD40L	>3781	53.58	76.74	0.646	0.0004
TNFβ	≤21.52	66.78	55.81	0.611	0.0214
TGFα	≤20.29	53.42	69.77	0.602	0.0347
IFNα2	≤246.45	89.69	20.93	0.512	0.7969
IL-13	>57.8	82.82	34.88	0.486	0.7654

2. Chemokines

MCP3	≤71.67	68.26	76.74	0.769	0.0001
IL-8	≤77.12	70.99	74.42	0.737	0.0001
MIG	≤6689	47.96	86.05	0.707	0.0001
IP-10	≤433.19	48.81	83.72	0.691	0.0001
MDC	>628	76.19	48.84	0.647	0.0004
MCP1	≤2752	78.23	53.49	0.637	0.0044
RANTES	>3371	94.88	32.56	0.605	0.0153
FRACTALKINE	≤253.21	51.7	72.09	0.591	0.0582
MIP1α	≤50.5	52.43	65.12	0.578	0.1049

MIP1β	≤99.89	76.79	34.88	0.534	0.483
GROa	≤89.57	69.07	44.19	0.529	0.5451
EOTAXIN	>259.32	55.1	55.81	0.506	0.905

3. Innate and adaptive growth factors

G-CSF	≤58.68	54.47	89.74	0.715	0.0001
GM-CSF	≤9.81	45.1	100	0.694	0.0502
M-CSF	≤927.52	72.4	53.66	0.655	0.0015
sIL-2Ra	≤979.22	44.86	79.07	0.646	0.0023
IL-2	≤3.73	76.42	42.86	0.578	0.1139
FLT3L	≤45.87	68.26	53.49	0.564	0.1864
IL-7	≤33.13	72.51	50	0.547	0.3381
IL-3	>1.39	32.69	77.78	0.53	0.6983

4. Adaptive cytokines

IFNγ	≤25.15	70.98	52.38	0.611	0.023
IL-17A	≤10.42	48.24	74.42	0.603	0.0327
sIL-4R	≤1909	72.11	51.16	0.59	0.0625
IL-17EIL-25	≤531.08	36.3	81.4	0.587	0.0715
IL-9	≤23.78	45.7	76.74	0.585	0.0786
IL-4	≤5.13	58.33	57.14	0.564	0.1886
IL-22	≤26.09	12.7	100	0.527	0.6511
IL-17F	≤168	82.46	50	0.508	0.9308
IL-5	≤57.38	92.86	13.95	0.507	0.8904
sCD30	≤72.53	16.76	93.33	0.502	0.9766

5. Wound-healing/repair growth factors

PDGFABBB	>63788	64.29	65.12	0.664	0.0001
FGF2	≤112.23	45.55	83.72	0.661	0.0007
EGF	>169.49	65.64	65.12	0.642	0.0006
PDGFAA	>8732	65.99	58.14	0.626	0.0029
sVEGFR3	≤1475	56.07	60	0.574	0.1388
sEGFR	≤48383	65.41	46.51	0.535	0.4663
VEGFA	>1385	27.55	83.72	0.53	0.5193
sVEGFR2	>12000	65.75	46.51	0.523	0.6264
sVEGFR1	>1227	16	100	0.469	0.6932

Table S3. Summary of soluble factors involved in the outcome of COVID-19 patients. This table represents a final summary of the most relevant cytokines that are elevated in patients who survive with respect to those who have a fatal outcome, and cytokines that are higher in patients who do not survive compared to those who survive.

Summary of soluble factors involved in the outcome of COVID-19 patients		
	**Survivors**	**Non-Survivor**
Functional cytokine group	Relevant differential increase of soluble factors	
**1. Proinflammatory and anti-inflammatory cytokines of the innate and innate/adaptive immune systems**		
**Innate**		IL-1α
		IL-1β
		IL-18
		IL-15
		IL-12p40
		IL-6
		IL-27
		IL-1RA
		sIL-1RI
		sIL-1RII
**Innate/adaptive**	sCD40L	TNFα
	TNFβ	TGFα
		IL-10
		sTNF-RI
		sTNF-RII
		sRAGE
**2. Chemokines**		
	MDC	IL-8
	RANTES	IP-10
		MCP1
		MCP3
		MIG
		FRACTALKINE

**3. Innate and adaptive growth factors**		
	G-CSF	M-CSF
	GM-CSF	sIL-2Ra
**4. Adaptive cytokines**		
		IFNγ
		IL-17A
		sIL-4R
**5. Wound-healing/repair growth factors**		
	EGF	FGF2
	PDGFAA	
	PDGFABBB	
